# Variability in the Length of Tail in the Dog

**Published:** 1896-08

**Authors:** M. Giovanolli


Variability in the Length of Tail in the Dog. By the same author. The author cites two singular cases: the first that of a bitch with her sixth litter of puppies; the mother had a short tail, but in each litter some of the young had long tails and others short ones. In the second case the mother had a tail of normal length; she was lined by a dog with a short tail; among the ten puppies only two had long tails.—*Ibid.*

